# Human Keratinocytes Are Vanilloid Resistant

**DOI:** 10.1371/journal.pone.0003419

**Published:** 2008-10-14

**Authors:** László Pecze, Kornélia Szabó, Márta Széll, Katalin Jósvay, Krisztián Kaszás, Erzsébet Kúsz, Tamás Letoha, János Prorok, István Koncz, András Tóth, Lajos Kemény, Csaba Vizler, Zoltán Oláh

**Affiliations:** 1 Institute of Biochemistry, Biological Research Center of the Hungarian Academy of Sciences, Szeged, Hungary; 2 Dermatological Research Group of the Hungarian Academy of Sciences, Szeged, Hungary; 3 Department of Medical Chemistry, University of Szeged, Albert Szent-Györgyi Medical and Pharmaceutical Center, Szeged, Hungary; 4 Department of Pharmacology and Pharmacotherapy, University of Szeged, Albert Szent-Györgyi Medical and Pharmaceutical Center, Szeged, Hungary; 5 Department of Dermatology and Allergology, University of Szeged, Albert Szent-Györgyi Medical and Pharmaceutical Center, Szeged, Hungary; 6 Acheuron Ltd, Szeged, Hungary; The Rockefeller University, United States of America

## Abstract

**Background:**

Use of capsaicin or resiniferatoxin (RTX) as analgesics is an attractive therapeutic option. RTX opens the cation channel inflammatory pain/vanilloid receptor type 1 (TRPV1) permanently and selectively removes nociceptive neurons by Ca^2+^-cytotoxicity. Paradoxically, not only nociceptors, but non-neuronal cells, including keratinocytes express full length TRPV1 mRNA, while patient dogs and experimental animals that underwent topical treatment or anatomically targeted molecular surgery have shown neither obvious behavioral, nor pathological side effects.

**Methods:**

To address this paradox, we assessed the vanilloid sensitivity of the HaCaT human keratinocyte cell line and primary keratinocytes from skin biopsies.

**Results:**

Although both cell types express TRPV1 mRNA, neither responded to vanilloids with Ca^2+^-cytotoxicity. Only ectopic overproduction of TRPV1 rendered HaCaT cells sensitive to low doses (1–50 nM) of vanilloids. The TRPV1-mediated and non-receptor specific Ca^2+^-cytotoxity ([RTX]>15 µM) could clearly be distinguished, thus keratinocytes were indeed resistant to vanilloid-induced, TRPV1-mediated Ca^2+^-entry. Having a wider therapeutic window than capsaicin, RTX was effective in subnanomolar range, but even micromolar concentrations could not kill human keratinocytes. Keratinocytes showed orders of magnitudes lower TRPV1 mRNA level than sensory ganglions, the *bona fide* therapeutic targets in human pain management. In addition to TRPV1, TRPV1b, a dominant negative splice variant was also noted in keratinocytes.

**Conclusion:**

TRPV1B expression, together with low TRPV1 expression, may explain the vanilloid paradox: even genuinely TRPV1 mRNA positive cells can be spared with therapeutic (up to micromolar) doses of RTX. This additional safety information might be useful for planning future human clinical trials.

## Introduction

Vanilloid receptor type 1 (VR1/TRPV1) is a member of the “transient receptor potential” (TRP) family of ion channels (20+) that show high levels of structural homology, particularly in the 6 transmembrane and ankyrin domain regions. TRPV1 is a Ca^2+^/Na^+^channel that, triggered by algesic endo-, and exovanilloids, moderate heat, inflammatory mediators and tissue acidification, transduces pain signals in the peripheral nervous system (PNS) [1]. TRPV1 is the cognate receptor of capsaicin (CAP), resiniferatoxin (RTX) and various other vanilloid-like analogous phytotoxins/irritants evolved as defense molecules in modern plants against mammalian herbivores. Contrary to mammals, avians, descendents of the dinosaurs can live a normal life without capsaicin sensitive V1 type TRP channel [2]. Indeed, avian seed dispersers are favored, whereas, mammalian herbivores are repelled by plants producing fruits with CAP or other toxic vanilloids [3].

TRPV1 positive nociceptors are an intermingled subset of primary sensory afferent neurons residing in cranial and spinal sensory ganglia [1]. From the ganglia TRPV1-immunoreactive fibers project to the sub-epidermis and intraepidermis in normal skin [4]. We have previously proposed that vanilloids, especially RTX, the most potent agonist of TRPV1, can be used to remove TRPV1^+^ neurons via specific Ca^2+^-cytotoxicity (i.e. necrosis) occurring within minutes, sparing other nociceptive neurons [5], [6], [7], [8], and the procedure had little or any side-effect in these mammals.

Expression of TRPV1 has recently been noted in the brain [9] and various non-neuronal tissues, such as pneumocytes, urothelium, gut epithelium, vascular endothelium, thymocytes, dendritic cells, mastocytes, smooth muscle, fibroblast and keratinocytes [10]–[12]. Interestingly, a number of these cell types claimed to be immunopositive to TRPV1 later turned out to be resistant to RTX. Vanilloid-elicited chemical knock-outs and TRPV1−/− mice do not show functional abnormalities other than loss of TRPV1-related moderate heat- and pain sensitivity [13], [14], except for somewhat impaired motility of the bladder [15]. Likewise, creating “chemical knock-outs” by either systemic CAP or RTX injection of newborn animals eliminates TRPV1 expressing neurons in the PNS, but beyond the neurological abnormalities noted in TRPV1−/− mice, no other gross abnormalities have been detected [16]. All these fact point to a lack of vanilloid-responsive TRPV1 in non-neuronal cell, while the eventual non-neuron-specific function of the receptor or its possible splice variants remains to be clarified.

Vanilloid sensitivity of non-neuronal cells is likewise a key question in the case of local vanilloid treatment. Topical application of capsaicin cream for the treatment or prurigo [17], HIV neuropathy [18] and other forms of neuropathies [19] was found to be an effective therapeutic intervention. A systematic review revealed that capsain, beside being the less expensive of the available therapeutic options, was among the most effective ones in treating post-herpetic neuralgia [20]. In the skin, beside the C-, and Aδ fibers, epidermal keratinocytes were also found to be TRPV1 immunopositive in a number of studies [21]–[23]. Our functional approach with both systemic and local RTX treatment, however, has determined these cells again to be resistant. In order to further address the vanilloid resistance paradox and to better characterize the treatment of topical application of capsaicin cream and RTX-mediated neurosurgery, we employed different diagnostic and functional assays in HaCaT cells [24], an immortalized human keratinocyte line. To compare and validate results in the established HaCaT cell line, some experiments were repeated in primary keratinocytes from skin biopsies and primary rat DRG culture.

To complicate functionality (i.e. vanilloid inducibility) of TRPV1 in keratinocytes, some previous reports claimed [21], [23], [25] but another contradicted the inductive function of vanilloids [26]. In addition to vanilloid resistance, we also addressed this contradiction in this paper. Neither our short (minutes scale) functional assays (^45^Ca^2+^-uptake, Co^2+^-uptake, of fluorimetric assays), nor long term (24 hr) cell survival assays showed keratinocytes to be responsive to TRPV1 mediated CAP/RTX effects. Nevertheless, cell survival assays show the non-TRPV1 mediated cell death elicited by high dose of CAP/RTX. The different concentrations of CAP/RTX between the TRPV1 mediated and non-TRPV1 mediated cell death clearly show that the therapeutic window is larger in the case of RTX. These results support our view that topical use of RTX containing creams may evolve into an effective pain relief option.

## Materials and Methods

### Reagents

Capsaicin (CAP), N-arachidonoyl-dopamine (NADA), agonists of TRPV1, capsazepine, a well characterized antagonist of TRPV1, and ionomycin (IONO), a calcium ionophore, were dissolved in DMSO (all from Sigma-Aldrich, St. Louis, MO). Anandamide (ANA) was obtained as an emulsion (in Tocrisolve®; Tocris Bioscience, Bristol, UK). Resiniferatoxin (RTX) (LC Laboratories, Woburn, MA) was dissolved in ethanol at 2 mg/ml concentration and further diluted in ddH_2_O. Propidium iodide (Sigma) was dissolved in PBS.

### Cells and cell culture

Normal human epidermal keratinocytes (NHEK) were derived from human skin samples obtained from esthetic surgery operations after obtaining written informed consent from the patients. The spontaneously immortalized human keratinocyte cell line HaCaT was kindly provided by Dr N. E. Fusenig, Heidelberg, Germany [27] and cultured in MIXMEM medium supplemented with 10% FCS (Sigma-Aldrich). The differentiation of HaCaT cells was induced by serum starvation as described [28]. Primary DRG cultures were prepared from E16 embryonic rats with method described previously [5] and slightly modified. Briefly, DRGs were dissected and then processed in Hank's balanced salt puffer until plated in DMEM. The DMEM contained 20 mM HEPES (to prevent acidification and stabilize pH at 7.4), 7.5% fetal bovine serum, 7.5% horse serum, 5 mg/ml uridine supplemented with 2 mg/ml 5-fluoro-2′-deoxyuridine, and 40 ng/ml nerve growth factor to inhibit cell division and to promote differentiation of long neuronal processes, respectively. Cells were seeded on 25-mm glass coverslips or on multi-well microtiter plates. Surfaces were coated with poly-D-lysine. The cultures were selected in this medium for 2 day, at which point well differentiated neurons and nondividing cells dominated the population. Human trigeminal ganglion neurons were collected from human cadavers after obtaining ethical clearance from the institutional ethical committee. The Sf9 insect cell line, derived from pupal ovarian tissue of the fall armyworm Spodoptera frugiperda and the HEK-293 human embryonal kidney cell line and NIH3T3 mouse fibroblast cell line were obtained from ATCC.

### Preparation of TRPV1-YFP and TRPV1 ε -expressing HaCaT cell line

The C-terminally tagged rat TRPV1ε DNA construct was prepared in the pMTH plasmid vector as described [5]. To avoid decrease of cell survival that occurs when TRPV1 is overexpressed, TRPV1ε was expressed using only the basal activity of the metallothionein (pMTH) promoter. The protein kinase C ε epitope tag allowed immunological detection. To prepare a cell line permanently expressing TRPV1ε, HaCaT cells were transfected with the pMTH-TRPV1ε plasmid using the Exgen 500 transfection reagent (Fermentas, Burlington, Canada). Transfections were carried out according to the recommendations of the manufacturer. After 24 h, cells were incubated with selection medium, containing 0.8 mg/ml G418 (Sigma-Aldrich). The selection medium was changed every second day. After 1 month, G418-resistant colonies were tested with vanilloid-induced ^45^Ca^2+^ uptake assays. A colony exhibiting capsaicin-induced ^45^Ca^2+^ uptake 20-fold above the base line determined in comparison with parental HaCaT cells was chosen for further studies.

### *^45^Ca^2+^* uptake assay

One day before the assay, cells were seeded in 96-well flat bottom plates (Orange Scientific, Braine-l'Alleud, Belgium) at a density of 20.000 cells/well. Assays were performed using a BioMek 1000 robotized liquid handler (Beckman Instruments, Fullerton, CA). The plates were washed three times with assay medium (Ca^2+^- and Mg^2+^-free Hanks' balanced salt solution supplemented with 0.8 mM MgCl_2_ and with 25 mM TRIS-HCl, pH 7.4). The agonist effect of ANA was measured at pH 5.5 because ANA is not active at pH 7.4 [29]. The dilutions of reagents containing ^45^Ca^2+^ were prepared using the robot. The ^45^Ca^2+^-uptake assay was performed for 10 min at room temperature using 1.33 µCi/ml of ^45^Ca^2+^ in 100 µl final volume/well. To stop ^45^Ca^2+^ uptake and remove the free isotope, cells were washed three additional times and then lysed in 80 µl/well lysis buffer (50 mM Tris-HCl, pH 7.5, 150 mM NaCl, 1% Triton X-100, 0.1% SDS, 5 mM EDTA) for 30 min. Seventy µl aliquots of the solubilized cell extracts were mixed with 100 µl aliquots of Optiphase Supermix scintillation cocktail (Perkin Elmer/Wallac Inc., Boston, MA) and counted in a Wallac Microbeta Trilux 1450 liquid scintillation counter (Perkin-Elmer).

### Vanilloid-induced Ca^2+^-cytotoxicity assay

Cells were seeded at 30 000 cells/well 24 h before adding RTX/CAP dilutions in duplicates or quadruplicates, then the plates were incubated for further 24 hr at 37°C. Cell survival was determined by the colorimetric MTS assay according to the instructions of the manufacturer (Promega, Madison, WI).

### Determination of changes in [Ca^2+^]_i_

HaCaT cells and primary keratinocytes were plated onto non-coated sterile glass coverslips at a density of ∼400.000 cells/cm^2^ and were investigated after 48 hours. Prior to the optical measurements, cells were incubated in culture medium containing 5 µM fluo-4 AM and 0.025% (w/v) Pluronic F-127 for 1 h, in dark, at room temperature. Subsequently, cells were washed in Tyrode medium for 10 min. Optical measurements were performed using an Olympus IX 71 inverted microscope (Olympus, Rungis, France). The coverslips were placed into a slotted bath chamber (37°C) at the microscope stage and cells were superfused with Tyrode alone for at least 10 min (control period). CAP (2 µM) was administered in the superfusion buffer for 10 min. At the end of the assay, *in situ* calibration was performed with the addition of 50 µM ionomycin. Changes in the intracellular free calcium [Ca^2+^]_i_ were determined by a single channel photon counting system. Cells (∼30/assay) in the 75*75 µm frame were illuminated at 485 nm and the emitted light was recorded at 535 nm. Raw data were recorded with HaemoSys (Experimetria, Hungary) and analyzed. The results were corrected with the decrease of fluorescence intensity in the case of untreated cells caused by bleaching and, probably, dye efflux due to the activity of efflux pumps.

### Cobalt histochemistry

TRPV1ε/HACaT, HaCaT and NHEK cells that had attached to the coverslips were washed in buffer A (in mM: NaCl, 57.5; KCl, 5; MgCl_2_, 2; HEPES, 10; glucose, 12; sucrose, 139; pH 7.4) for 2 min. Cells were then incubated at 37°C in cobalt-uptake solution (buffer A+5 mM CoCl_2_) containing 50 µM RTX for 10 min. Following a brief wash in buffer A, the water-soluble cobalt taken up by the cells was precipitated by 0.12% ammonium polysulphide (Sigma-Aldrich) put into buffer A, which resulted in the formation of dark, water-insoluble CoS in TRPV1 positive cells. Cells were fixed in 4% formaldehyde and mounted on glass slides using Kaiser's Glycerol-Gelatine (Merck, Darmstadt, FRG). Cells were examined by a Nikon light microscope (Melville, NY).

### Synchronization of HaCaT keratinocytes, and detection of TRPV1 transcript level

Briefly, HaCaT keratinocytes were synchronized by contact inhibition and serum starvation as it was described in details in a previous publication [30]. The culture was released from the quiescent state by passaging (0 h) and grown for 168 h. The various states of HaCaT keratinocyte proliferation and differentiation were monitored by propidium-iodide staining (data not shown). Samples for mRNA expression studies were taken at time points 0, 12, 24, 36, 48, 72, 96, 168 h, and total RNA was purified by TRIzol reagent following the instructions of the manufacturer (Invitrogen, Carlsbad, CA, ). cDNA was generated with oligo(dT) and random hexamer primers from 1 µg total RNA, using the iScriptTM cDNA synthesis kit of Bio-Rad Laboratories (Hercules, CA, USA) in a final volume of 20 µl. Real-time RT-PCR experiments were performed to quantify the abundance of TRPV1 and TRPV1b splice variants. Primers and probe specific for human TRPV1 were: 5′-GTGCACTCCTCGCTGTACGA-3′ and 5′-CACCTCCAGCACCGAGTTCT-3′ forward and reverse primers respectively, and the TaqMan probe used was 5′-FAM-TGTCCTGCATCGACACCTGCGAG-TAMRA-3′. Primers and probe specific for human TRPV1b were: 5′-GAATGACGCCGCTGGCT-3′ and 5′-CAGCGGCRCCACCAAGAG-3′ forward and reverse primers respectively, and the TaqMan probe used was 5-′FAM-GGGAAGATCGGGAATCGCCACGA-TAMRA-3′ [31]. Real-time RT-PCR reactions were performed using iQ Supermix and an iCycler (Bio-Rad Laboratories).

Data were compared using repeated measures (ANOVA) followed by Dunnett's post hoc test test to determine statistical differences after multiple comparisons (STATISTICA). Probability values of less than 0.05 tests were considered significant, and all data was compared to day 0 values.

### Induction of differentiation of normal human keratinocytes by Ca^2+^-treatment

Normal human epidermal keratinocytes were isolated and cultured as described previously [32]. Briefly, human epidermal cells were obtained from healthy individuals undergoing plastic surgery after informed consent according to Institutional Review Board protocol. After removal of the subcutaneous tissue and much of the reticular dermis, the tissue samples were cut into small strips and incubated in Dispase® solution (grade II; Roche Diagnostics, Mannheim, Germany) overnight at 4°C. On the following day, the epidermis was peeled off the dermis, and was incubated in 0.25% trypsin solution (Sigma-Aldrich) at 37°C for 30 min and aspirated using a Pasteur pipette to aid cell dissociation. The viability of the cells was always >95% as determined by Trypan blue exclusion. A suspension of primary epidermal cells was prepared in keratinocyte serum-free medium (Keratinocyte SFM, Invitrogen; supplemented with antibiotic/antimycotic solution, Sigma-Aldrich) at a density of 4×10^4^ cells/cm^2^ in 75 cm^2^ tissue culture flasks (Corning, Corning, NY). Human epidermal keratinocytes were cultured in Keratinocyte-SFM in a humidified atmosphere containing 5% CO_2_. The medium was changed every 2 days.

In third passage, the cells were grown up to 80–90% confluence in 25 cm^2^ tissue culture flasks (Corning). At this point (day 0) they were fed with calcium-free keratinocyte SFM (GIBCO/BRL and Invitrogen), supplemented or not with 1.7 mM CaCl_2_. Elevation a of the calcium level in culture has been shown to induce differentiation of human keratinocytes [33]. The culturing media was changed on day 1, 2, 4, 6, 8, 10, when samples were also taken for mRNA expression experiments. RNA was isolated, cDNA was generated, and real-time RT-PCR for the detection of TRPV1 splice variants were performed as described for HaCaT keratinocytes. The differentiation process was monitored by following the involucrin mRNA expression of the cells using real-time RT-PCR (data not shown). Involucrin, a major protein of the cornified envelop was defined as marker for keratinocyte terminal differentiation [34].

### Protein Extraction and Western Blot

Human trigeminal ganglions were excised from cadavers after obtaining the proper ethical clearance from the institutional ethical board of the University of Szeged. Tissue samples were homogenized in modified RIPA buffer (50 mM Tris-HCl, 140 mM NaCl, 5 mM EDTA, 1% TritonX-100, Protease Inhibitor Coctail - Roche) and incubated on ice for 15 minutes to let lysis proceed. All samples were precleared by centrifugation (15′ 12000 g at 4°C) before determining protein concentrations using the BCA method (Sigma). PAGE was done as described in the Protein Electrophoresis technical manual of Amersham Biosciences. Protein samples were separated on 8% polyacrylamide gels, then transferred to Millipore Immobilon PVDF membrane using Tris-Glycine transfer buffer (0.192 M Glycine, 25 mM Tris, 20% MetOH). Transfer was followed by blocking of the membrane (30′ at RT in 5% dry milk TBS-T), incubation with primary antibody (overnight at 4°C, in 0.5% BSA TBS-T), secondary antibody (2 h at RT, in 1% dry milk TBS-T), results were revealed using the ECL method (SuperSignal West Chemiluminescent Substrate - Pierce, Hyperfilm ECL - Amersham). TBS: 50 mM Tris, 140 mM NaCl, pH 7.6; TBS-T: TBS with 0.5% Tween-20. Antibodies used and their dilutions: TRPV1 - 1∶1000 (ABR Affinity Bioreagents #PA1-748), ß-actin - 1∶1000 (Sigma #A5060), and anti-rabbit HRP - 1∶10000 (Sigma #A6154, respectively).

### Flow cytofluorometry

For assessing the dependence of vanilloid cytotoxicity on the expression level of the TRPV1 receptor, 3T3 fibroblasts stably expressing variable levels of an YFP-tagged TRPV1 construct were incubated with 0.1, 1 or 10 µM capsaicin for 30′ then propidium iodide was added at 3 µM end concentration and capsaicin-induced necrosis was detected with flow cytofuorometry.

## Results

**Figure 1:**
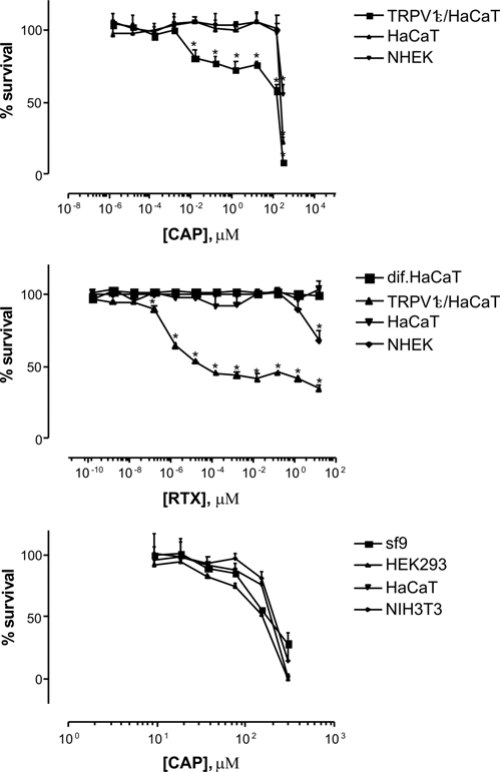
Keratinocytes were not killed by low dose vanilloids. Primary human keratinocytes (NHEK), proliferating and differentiating HaCaT keratinocytes, TRPV1-expressing HaCaT keratinocytes, HEK293 human embryonal cells, NIH3T3 mouse fibroblasts and sf9 insect cells were grown for 24 hr on 96-well plates in the presence of indicated concentrations of RTX (A) and CAP (B, C). Cell survival was evaluated by the MTS bioassay. Only HaCaT cells stably transformed with TRPV1 transgene were sensitive to cytotoxicity triggered by low dose vanilloids, while normal keratinocytes were only killed by extremely high vanilloid doses, likewise toxic to the negative control insect cells, HEK293 or NIH3T3 cells. Data from a representative experiment repeated three times with similar results is shown. Values are shown as means±SD. (*: p<0.05, t-test).

When exposed to RTX/CAP, cells expressing functional TRPV1 receptor undergo a rapid elevation of [Ca^2+^]_i_, leading to membrane disruption and exceptionally rapid necrosis occurring within minutes. Taken into account the very rapid cytotoxic effect, we have chosen a 24 h colorimetric cell survival assays for assessing the presence of a classic TRPV1 receptor on RTX-treated primary keratinocytes, normal and differentiated HaCaT cells, as well as on HaCaT cells transformed with TRPV1. Only the TRPV1-transfected HaCaT cells were sensitive to TRPV1-dependent cytotoxicity induced by vanilloids applied at pharmacological concentrations, corroborating our hypothesis on the lack of functional TRPV1 receptor. Normal human epidermal keratinocytes were sensitive only to very high concentrations of RTX. This concentration (16 µM), however, is very close to that (50 µM) established in a study describing a non-TRPV1 mediated cytotoxic action of vanilloids [35] (Fig. 1A). Each keratinocyte type was sensitive to the highest concentration of capsaicin (300 µM)(Fig. 1B). This capsaicin concentration, 300 µM, was equally toxic to avian cells expressing a well-described capsaicin-insensitive TRPV1 [2], and even to insect cells (the sf9 cell line), obviously negative for a mammalian type TRPV1 (Fig. 1C.).

**Figure 2:**
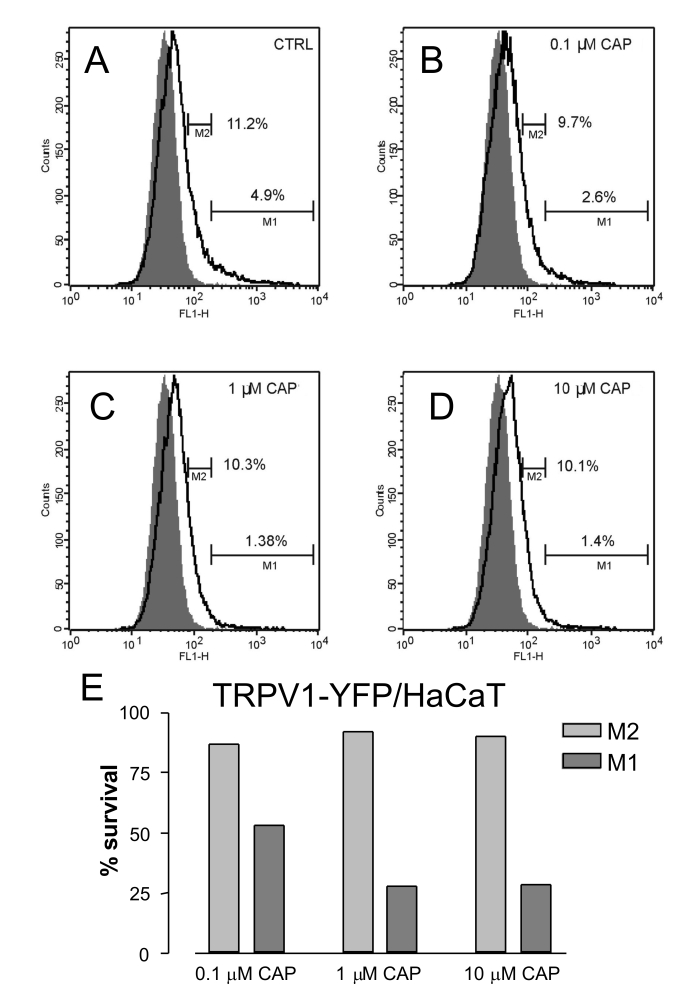
Transformed cells expressing low levels of TRPV1 were resistant to TRPV1-mediated capsaicin toxicity. NIH3T3 mouse fibroblasts expressing variable levels of TRPV1-YFP fusion protein were incubated in the absence of capsaicin (A) or with different capsaicin concentrations, 0.1 µM (B), 1 µM (C), 10 µM (D) for 30′, then propidium iodide was added and the flow-cytofluorometric analysis was performed. The M1 and M2 regions contain the intact (propidium iodide negative) TRPV1-YFP high and low positive cells, respectively. Only TRPV1 high positive cells were sensitive to dose dependent, TRPV1 mediated killing by capsaicin (E).

Using flow cytofluorometry we assessed how the cytotoxic effect of TRPV1 agonists depends on the TRPV1 expression level and concentrations of agonists. Capsaicin applied at pharmacological concentrations only killed HaCaT cells expressing the TRPV1-YFP fusion protein above a certain threshold level, while low TRPV1 positive cells were resistant to the applied capsaicin concentrations, 0.1–10 µM (Fig. 2).

**Figure 3:**
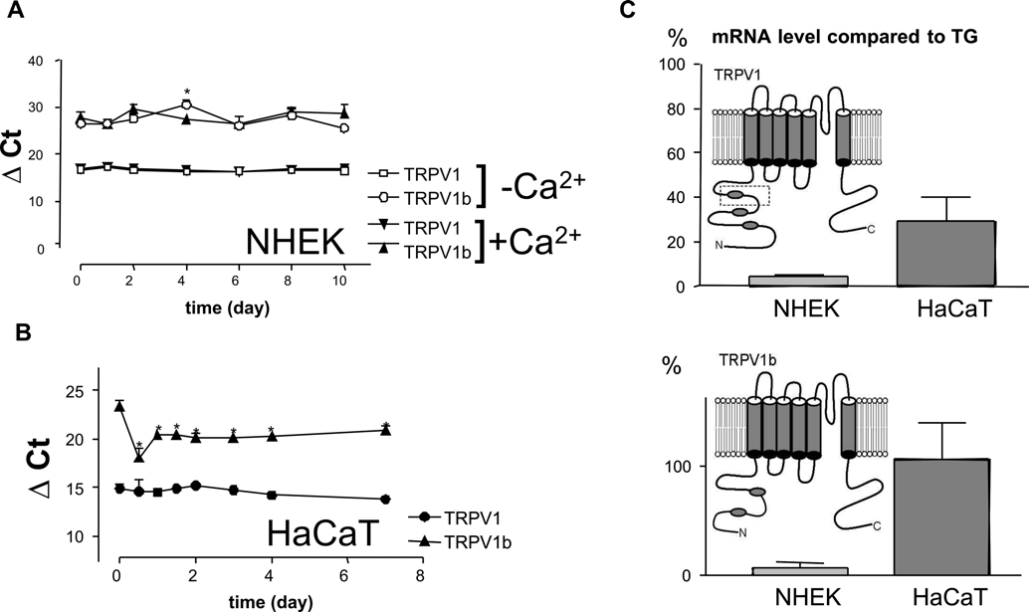
Keratinocytes express low levels of the full length TRPV1 transcript together with transcript of the TRPV1b dominant negative splice form. Detection of TRPV1 transcript in differentiating human cultured keratinocytes (NHEK) (A) and HaCaT cells (B) by real-time RT-PCR. TRPV1 threshold cycle numbers normalized to the internal control 18S rRNA (delta CT) are plotted. The amount of mRNAs is inversely proportional to the plotted threshold cycle numbers. Data are presented as means of three independent experiments. (*: p<0.05, Dunnet test). The amount of mRNA was also compared in samples from cultured keratinocytes, human HaCaT cells and human trigeminal ganglion (C). The schematic model of TRPV1 and TRPV1b is shown in the corresponing graphs.

**Figure 4:**
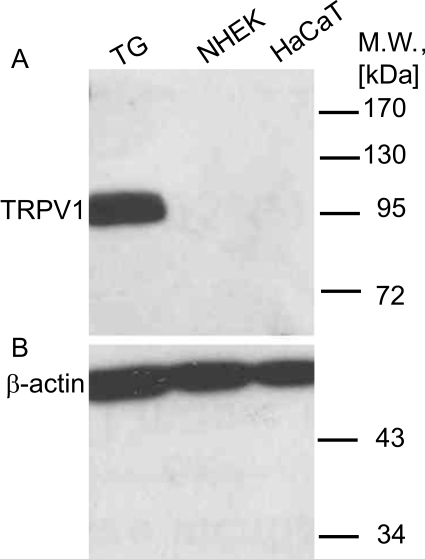
The TRPV1 protein level in the keratinocytes was under detection limit in Western blotting assay. Proteins were extracted from human trigeminal ganglion (TG), primary human keratinocytes (NEHK) and the HaCaT cell lines. With a commercially available polyclonal antibody only the TG expressed 94 kDa TRPV1 can be detected (A). The equal loading of protein extracts was validated by β-actin (∼50 kDa), a common, ubiquitously expressed cytoskeletal protein (B).

We detected the presence of both TRPV1 and the dominant negative TRPV1b transcripts by real-time RT-PCR using primers and TaqMan probes specific either to TRPV1 or TRPV1b transcripts. The abundance of TRPV1 compared to TRPV1b was obvious as it was detected in other tissues [31]. The Ca^2+^ induced differentiation of cultured keratinocytes was accompanied with slight changes at the level of both transcripts (Fig 3A) with differences among various donors, depending on the level of differentiation in the adjacent cultures that was monitored by involucrin expression (data not shown). While only slight changes were detected in the transcript level of TRPV1 after HaCaT cells exited the quiescent state and serum induced cell division occurred (Fig 3B), the level of TRPV1b transcript increased significantly and remained elevated throughout the experiment. The point-by-point statistical comparison of TRPV1b transcript levels revealed that the expression was significantly elevated at all time points compared to day 0. The gross differences of deltaCT values of TRPV1 and TRPV1b both in keratinocytes and HaCaT cells suggest thirty-, and one thousand-fold differences in transcript levels, respectively. The amount of mRNA was compared in samples from cultured keratinocytes, human HaCaT cells and human trigeminal ganglion. The level of the TRPV1 transcript in keratinocytes was orders of magnitudes lower than that in trigeminal ganglion. The level of TRPV1b transcript was approximately the same both in human trigeminal ganglion and in HaCaT (Fig. 3C). Unlike its mRNA, TRPV1 protein was detectable only in samples extracted from human trigeminal ganglion (TG). (Fig. 4).

**Figure 5:**
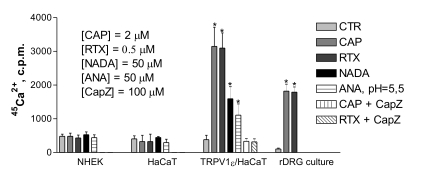
Neither endo-, nor exo-vanilloids caused ^45^Ca^2+^-influx in keratinocytes. Primary human keratinocytes (NHEK), primary culture of DRG neurons, HaCaT keratinocytes and TRPV1 transformed HaCat keratinocytes were seeded in 96-well plates, then incubated for 10-min with different TRPV1 agonists (capsaicin, CAP; resiniferatoxin, RTX; N-arachidonoyl-dopamine, NADA; Anandamide, ANA) and combinations of agonists and antagonists (CAP or RTX+capsazepine, capZ) in the presence of ^45^Ca^2+^. Cell-bound radioactivity was measured with liquid scintillation. The bars represent mean scintillation counts per minute (c.p.m.)+SD of eight parallel samples from a representative experiment repeated three times with similar results.

To further test vanilloid sensitivity, we determined the endo- and exovanilloids-induced ^45^Ca^2+^-uptake of keratinocytes and rat DRG neurons in a robot-based bioassay. Neither primary keratinocytes nor HaCaT cells showed ^45^Ca^2+^ influx in response to vanilloids. On the other hand, a HaCaT cell line established to express TRPV1 ectopically and primary DRG culture readily showed ^45^Ca^2+^ influx upon challenge with these agonists. The used concentrations of agonists (CAP 2 µM, RTX 0.5 µM, N-arachidonoyl-dopamine 50 µM, Anandamide 50 µM at pH 5.5) were higher than the half-effective concentrations for both rat and human TRPV1 receptor determined by us or found in literature [29], [36], [37]. The ^45^Ca^2+^-influx was completely abrogated by capsazepine (IC_50_≈10 µM at 2 µM CAP), a bona fide channel blocker of TRPV1 (Fig 5). Differentiated HaCaT cells were also found to lack capsaicin-induced or RTX-induced Ca^2+^-uptake (data not shown). Our genetic construct contains a Zn^2+^-inducible promoter, therefore, TRPV1 could be expressed at different levels. Higher levels positively correlated only with increased V_max_, and not with EC_50_ of the agonists (EC_50_ 2.6 nM, RTX and 70 nM, CAP) in Ca^2+^-influx assays.

**Figure 6:**
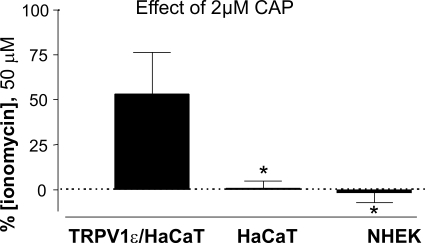
Fluorometric Ca^2+^ imaging showed no capsaicin-induced Ca-signal in keratinocytes. Primary human keratinocytes and HaCaT cells were loaded with Fluo4-AM Ca^2+^-indicator then imaged in an appropriately equipped fluorescence microscope setup. The bars represent the increase in fluorescence upon capsaicin (2 µM) treatment as a % of the fluorescence increase after ionomycin treatment (50 µM). While TRPV1 transformed cells exposed to CAP showed readily detectable fluorescence changes, neither untransformed HaCaT cells, nor primary keratinocytes responded to this algesic vanilloid substance. Values are shown as means±SD for 6 measurement. The significance of differences compared with TRPV1ε/HaCaT was determined with the paired t-test, *P<0.05.

Fluorimetric Ca^2+^ imaging assays were carried out with indicator dye-loaded cells that can reflect subtle changes in [Ca^2+^]_i_, with higher sensitivity than either isotope influx determination in high number of cells or agonist-induced cytotoxicity quantified by photometric methods. Both primary human keratinocytes and HaCaT cells were tested after preloading with Fluo4 in an imaging setup. While TRPV1 transfected cells exposed to CAP showed readily detectable fluorescence changes, neither normal HaCaT cells, nor primary keratinocytes responded to this algesic vanilloid substance, whereas ionomycin treatment of cells showed that the test system is functional and Ca^2+^ signal can be detected (Fig. 6).

**Figure 7:**
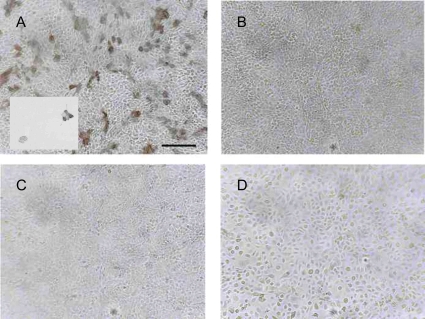
Cobalt histochemistry did not show functional TRPV1 in keratinocytes. A: TRPV1ε/HaCaT and rat DRG culture (insert) treated with 50 µM RTX in the presence of cobalt cations shows dark CoS precipitate. B: TRPV1ε/HaCaT without RTX C–D: HaCaT and NHEK treated with 50 µM RTX did not produce the characteristic precipitate formed in the presence of intracellular cobalt cations. Bar represents 0.2 mm.

Previous to discovery of TRPV1 capsaicin-induced Co^2+^-uptake was routinely used to identify inflammatory pain nociceptive neurons by histochemistry [10], [38]–[41]. In our study we found positive staining of TRPV1ε/HaCaT and rat DRG primary culture cells treated with RTX. Non-transfected HaCaT and NHEK cells didn't show any TRPV1 positive staining (Fig. 7).

## Discussion

Although its mechanism of action is debated, capsaicin is used as a topical analgesic for various cutaneous disorders [42], [43]. It is thought that prolonged repetitive applications of capsaicin depletes substance P (SP) and other neuropeptides existing in the small-diameter afferent fibers [44], and then desensitizes TRPV1 receptor/cation channel [45]. Although transient, functional desensitization induced by low dose vanilloids does exist [46], [47], we think that the pain relieving effect of vanilloids, i.e., extremely high doses of capsaicin or lower doses of the more potent vanilloid RTX are not based on desensitization based on signaling events, generally transient and reversible. One of us earlier proved that vanilloids may specifically ablate primary afferent nociceptors [6]. In accordance with this, systemic RTX-treated animals remained insensitive to vanilloids in their whole life. In contrast, a lower dose of RTX injected in the paw causes only a reversible inactivation (3 weeks) of nerve endings removing only the vanilloid sensitive axons remote to the neuronal cell body [6], allowing for of axon regeneration. Importanly, even when TRPV1 positive neurones were deleted, no non-neuronal side effects were detected. Validation of vanilloid resistance of non-neuronal cell types would provide important safety data, expanding the currently claimed potential of the molecular neurosurgery technology.

Here we showed that RTX did not harm keratinocytes, suggesting that capsaicin can be replaced in the dermatological practice as soon as this agent will be registered as drug. Due to better therapeutic window RTX can be applied topically to nerve endings in the skin to alleviate neuropathic pain and severe inflammations.

We validated here with different experimental means the previous hypothesis that TRPV1 mRNA expressed both in proliferating and in differentiated keratinocytes as well as in HaCaT cells does not confer agonist-mediated increase in [Ca^2+^]_i_ and subsequent cell death, analogous to that observed during TRPV1+ sensory neuron depletion. These results draw attention again to a paradox that have recently been generated by a number of conflicting observations from other laboratories working with various diagnostic means to characterize molecular phenotype, such as screens of TRPV1+ in various cells and tissue types.

Vanilloids in high concentrations, due to distant homology to flavonoids, important co-factors in various red/ox systems, induce apoptosis in number of different cell types by causing either mitochondrial depolarization [35], or inhibition of electron transport in the plasma membrane [48]. Thus, high doses of vanilloids (i.e. 20–100 µM) do not require expression of TRPV1 for the cytotoxic effect. In fact, we could elicit non-TRPV1 mediated cell loss with [RTX]>16 µM and [CAP]>200 µM, respectively, to demonstrate this phenomenon. Under the noted threshold values, however, these vanilloids were harmless to cells lacking TRPV1 expression. The dose of RTX causing non-TRPV1-mediated cytotoxicity was ∼10^4^ fold higher than that (1 nM) inducing TRPV1-dependent Ca^2+^-cytotoxicity in *bona fide* DRG sensory neurons [5] and ∼10^8^ fold higher than the dose (0.1 pM) necessary to induce Ca^2+^-cytotoxicity in a cell line ectopically overproducing TRPV1. Our results obtained in insect cells, an obviously TRPV1 negative control, also demonstrated the lack of receptor specificity of high-dose vanilloids. The dose of CAP concentrations causing non TRPV1 mediated cytotoxicity was ∼10^5^ fold higher than the dose (1 nM) necessary to induce TRPV1-dependent cytotoxicity in TRPV1 transfected cell lines. All together, this means that RTX has a broader therapeutic window than CAP, making it a more desirable candidate for human clinical application.

We showed evidences that agonist-dependent, receptor-mediated cytotoxic effect requires a threshold-level expression of TRPV1. Prolonged exposure to relatively high concentration of RTX (i.e. low micromolar) is definitely tolerated by keratinocytes in cell culture. Levels of TRPV1 transcript in keratinocytes, however, were orders of magnitudes lower than in human trigeminal ganglion neurons. Conceivably, TRPV1 protein expression below the threshold required for vanilloid sensitivity may explain unresponsiveness to vanilloids.

However, other factors may also contribute to vanilloid insensitivity. TRPV1 is not the only member of the TRP family of ion channels. Functional TRPV1 is most likely a homotetramer [49], although the existence of hetero-multimers of TRPV1 with other members of the TRPV subfamily is possible. TRPV1 is reportedly co-expressed with other isotypes with variable stochiometry in keratinocytes and other vanilloid resistant cells. For example, TRPV2 co-expression was reported in rat DRG and brain [50], and TRPV4 in a subset of nociceptive neurons where it sensitizes effects of prostaglandin PGE_2_ in inflammatory thermal hyperalgesia [51]. These isotypes obviously do not have dominant negative effect in C-type sensory neurons localized in ganglia of the peripheral nervous system or at least not at that stochiometry and levels they are expressed. Occurrence of TRPV3 and TRPV4 has been reported in keratinocytes; TRPV4 in suprabasal keratinocytes, whereas TRPV3 selectively in the basal layers of the epidermis [52], [53]. The presence of these receptors might have interfered with heat sensitivity assays assessing TRPV1 functionality in earlier studies. Their different chemical sensitivity makes these TRP receptors easily distinguishable from TRPV1. Importantly, due to their different ligand specificity, they might not play a role in the response to transdermal vanilloids. On the other hand, TRPV1 might be capable of forming hetero-oligomeric complexes either with TRPV3 or TRPV4, as demonstrated in ectopic expression systems [54], yet functionality of these receptor/channels not entirely known. Co-localization of TRPV1 and TRPV1b, its splice variant in the same cell confers a dominant negative effect and receptor inactivation in the nervous system as well as in other tissues [31] and this rule may apply to TRPV1+ keratinocytes, in which we determined unequivocal presence of TRPV1b. Alternatively, resistance to vanilloids can appear due to lack of cyclin dependent kinase 5 signal transduction pathway, a protein kinase upstream to TRPV1 that might also necessary for channel opening [55].

Last but not least, the discrepancy between our results and observation of others' could be caused by false positive signals produced by antibodies that not entirely specific to TRPV1 and can cross-react with other TRP isotypes. Commercially, up till now, only polyclonal antibodies were available that prepared against peptide antigens. Linear epitopes frequently results in antibodies of suboptimal specificity and selectivity and not even ideal for semi-quantitative determination of the TRPV1 protein. Dendritic cells seem to be an example of such false positive determination of TRPV1 [56], [57]. Likewise, in a number of other papers, keratinocytes show negative TRPV1 immuno-staining, such as immuno-positive nerve endings surrounded by apparently unstained keratinocytes, suggesting that their anti-peptide polyclonal TRPV1 antibody does not recognize the protein (or its tissue-specific splice form) in non-neuronal cells [4], [58], [59].

To fully characterize functionality, we have to take into consideration that there are two pools of TRPV1 in a cell: one in the plasma membrane (TRPV1_PM_) and the other in the endoplasmic reticulum (TRPV1_ER_) [5]. ^45^Ca^2+^- or Co^2+^-uptake assays can reveal the function of TRPV1_PM_ alone, while, flourometric Ca^2+^-imaging depicts the combinations of the TRPV1_PM_ and TRPV1_ER_ receptor populations. In keratinocytes we neither have found vanilloid-induced TRPV1 mediated response in our short term ^45^Ca^2+^- or Co^2+^-uptake assays nor any positive evidence of intracellular free Ca^2+^ mobilization. Previous studies with comparable means demonstrated that a minor fraction of keratinocytes may exhibit a CAP-induced fluorometric Ca^2+^ influx signal similar to that demonstrated by one of us in TRPV1^+^ DRG neurons. However, majority of the population showed a rather spontaneous Ca^2+^ signal, again a typical biomarker of false positive membrane activity, neutral to either presence or absence of a vanilloid agonist [21], [23].

In whole-cell voltage clamp functional bioassays carried out in mouse keratinocytes CAP has evoked little if any Ca^2+^-response, even concentration raised up to 10 µM (i.e. CAP's ED_50_ = 0.3 µM in TRPV1+ DRG neurons) [26] Nevertheless, the CAP-evoked intracellular Ca^2+^ increase is not a specific marker for TRPV1 function, especially if applied in high doses. TRPV1 negative cell lines, such as activated T-lymphocytes, and Jurkat cells show readily intracellular Ca^2+^-elevation upon treatment with 200 µM CAP [48], obviously, a mechanism distinct of vanilloid-induced receptor mediated cation uptake. Endogenous activation of TRPV1 has been suggested to lead to Ca^2+^-dependent production of proinflammatory mediators such as prostaglandin E2 and interleukin-8 [21]. Our experiments in keratinocytes suggest that the increase of pro-inflammatory mediators is attributable to a TRPV-independent, yet not fully characterized cell necrotic pathway.

Our validated, high sensitivity and specificity bioassays proved keratinocytes completely vanilloid resistant. Importantly, the functional TRPV1 in keratinocytes could have made them a problematic target seemingly able to generate unwanted side effects and prevent future use of either transdermal capsaicin or RTX. Based on our data here, together with the lack of effect in skin determined in knockout or RTX-treated mice *in vivo* [13], [14] keratinocytes can be safely spared from vanilloid-induced, receptor-mediated cytotoxicity and cell death, even when the most potent agonist RTX will be employed in molecular neurosurgery as proposed previous to these studies. The skin side effects of transdermal capsaicin observed in clinical practice thus seem to be entirely caused by the affected sensory nerves, especially to to the CGRP and substance P liberated in an entirely TRPV1- and sensory nerve dependent manner, not separable from the therapeutic effect. Two approaches might be envisageable for alleviating these side effects – one is substitution of capsaicin with more potent vanilloids that would rapidly kill the sensory nerves, thus paradoxically decreasing the pain sensation and local inflammation. The second is concomitant use of local anesthetic agents (lidocain) acting downstream of the TRPV1 receptor. [18]

It is important to note that, in spite of the assumed presence of TRPV1 receptor in the brain [9], [60], [61], capsaicin (100 nM–100 µM) and RTX (100 nM) failed to alter the basal intracellular Ca^2+^-levels in the rat hippocampal nerve terminals [62]. Likewise, the presence of TRPV1 transcript as shown here and reported in cases of other cell types not necessarily means sensitivity to potent vanilloid phytotoxins. Immunoreactivities that have recently reported in number of diverse cell types and identified in various central nervous system neurons can be misleading and are only just another false positive biomarker of TRPV1. Since our Western-blotting using protein extract from keratinocytes was negative to TRPV1, we can not exclude the possibility that keratinocytes do not express TRPV1 at a meaningful protein level at all. Therefore, study of functionality in addition to diagnostics is needed for better understanding of the *in vivo* status of TRPV1. All together these results may explain the relatively modest neural/non-neuronal side effects of the RTX-mediated molecular neurosurgery that systemically can create “chemical knockout” with lack of major behavioral deviances. Our observations here are also corroborated by the lack of obvious phenotype in TRPV1−/− mice.
